# The Effect of Chronic Graft-Versus-Host Disease and Its Therapy on Salivary Caries Risk Factors—An Exploratory Cross-Sectional Pilot Study †

**DOI:** 10.3390/healthcare13243265

**Published:** 2025-12-12

**Authors:** Nina Vovk, Manca Urek, Ksenija Cankar, Lidija Nemeth

**Affiliations:** 1 Department of Dental Diseases and Normal Dental Morphology, Faculty of Medicine, University of Ljubljana, 1000 Ljubljana, Slovenia; nina.vovk@mf.uni-lj.si; 2 Dental Center Osovnikar, 4220 Škofja Loka, Slovenia; manca.urek@gmail.com; 3 Institute of Physiology, Faculty of Medicine, University of Ljubljana, 1000 Ljubljana, Slovenia; ksenija.cankar@mf.uni-lj.si; 4 Division of Stomatology, University Medical Centre Ljubljana, 1000 Ljubljana, Slovenia

**Keywords:** chronic graft-versus-host disease, dental caries, saliva, salivary flow, cyclosporine, extracorporeal photopheresis

## Abstract

**Background**: The aim of this study was to investigate the effects of chronic graft-versus-host disease (cGVHD) and its treatment with cyclosporine and extracorporeal photopheresis (ECP) on salivary caries risk factors. **Methods**: In this exploratory single-centre cross-sectional pilot study, saliva samples from 22 cGVHD patients were analysed for flow rate, pH, buffering capacity, and counts of *Streptococcus mutans* and *Lactobacillus*. A detailed dental examination assessed plaque, carious lesions, and their progression. Caries risk was determined based on general health and diet questionnaires and clinical findings. **Results**: Patients receiving a combination of cyclosporine and ECP had significantly fewer carious teeth, affected tooth surfaces, and non-cavitated carious lesions compared with those treated with ECP alone (Bonferroni test, *p* = 0.004, *p* = 0.002, and *p* < 0.001, respectively). Patients treated with ECP had more carious teeth and affected surfaces than those who did not receive either ECP or cyclosporine (*p* = 0.008 and *p* = 0.002), whereas patients treated with cyclosporine only had more non-cavitated lesions than those receiving both cyclosporine and ECP (*p* < 0.001). A negative correlation was observed between cyclosporine dose and stimulated salivary flow (R = −0.672, *p* = 0.0486), and a positive correlation between cyclosporine dose and caries risk (R = 0.640, *p* = 0.0461). **Conclusions**: The disease and its treatment were associated with reduced salivary flow and increased caries risk. Patients’ oral health should be monitored regularly and managed with care to prevent further deterioration.

## 1. Introduction

Chronic graft-versus-host disease (cGVHD) is a serious immune-mediated complication following allogeneic haematopoietic stem cell transplantation (allo-HSCT) for various blood disorders [1]. The pretransplant conditioning regimen aims to eliminate the underlying disease and modulate the immune system to facilitate engraftment [2]. The disease develops in up to 80% of transplant recipients. It occurs in 20–40% of paediatric patients and in approximately 50% of adults, with the prevalence increasing to around 60% among older allo-HSCT recipients [3,4,5]. The disease is characterised by heterogeneous inflammation, tissue damage and fibrosis, and typically develops between 3 months and 2 years after transplantation [6]. Diagnosis is based on distinctive clinical manifestations across multiple organ systems, which mimic different autoimmune diseases, such as scleroderma or Sjögren syndrome [7]. According to the NIH consensus criteria, the disease is categorised as mild, moderate, or severe based on the number of organs involved and the degree of functional impairment in each affected organ [6].

According to the European Society for Blood and Marrow Transplantation, steroids (prednisone) alone or in combination with calcineurin inhibitors (CNI) are used as first-line treatment. In patients with mild cGVHD, topical immunosuppressants (IS; steroids, CNI, or phototherapy) are usually sufficient, as organ function is not impaired. If topical treatment is inadequate, systemic IS should be administered alone or in combination with topical IS. For moderate or severe cGVHD, systemic steroids are used. In cases of severe cGVHD, CNI (cyclosporine or tacrolimus) are usually combined with steroids to reduce the steroid dose [8]. Extracorporeal photopheresis (ECP), an immunomodulatory therapy, is a commonly used second-line therapy for patients with cGVHD who do not respond to corticosteroids [9,10,11].

The oral cavity is one of the most affected sites in cGVHD (45–83% of cases) [12,13,14,15]. Conditioning regimens, disease progression and immunosuppressive therapy lead to deterioration of the salivary gland acini, altering both the composition and volume of saliva [13,16]. Clinical manifestations commonly include reduced tongue mobility, trismus, mucosal alterations, taste disturbances, and salivary gland dysfunction with hyposalivation and xerostomia [17]. Oral cGVHD usually does not respond to systemic treatment of skin, liver, or eye manifestations with steroids and immunomodulatory drugs. Complementary local therapies are used to alleviate oral symptoms, including gels, various mouthwashes and sprays, CO_2_ laser therapy, and photobiomodulation [17,18,19,20].

Saliva plays a crucial role in oral homeostasis through continuous cleansing of oral surfaces, acid neutralisation, and antimicrobial activity. However, this homeostatic function can be compromised when salivary flow diminishes, leading to various oral problems including a high risk of caries [18,21,22,23,24]. The chronic and complex nature of caries is strongly affected by disruptions in normal salivary function, affecting either its volume or composition [21].

Dental caries, an infectious multifactorial disease, involves the demineralisation of hard dental tissues by acids produced by cariogenic bacteria, which lower the pH of the oral cavity [25]. A carious lesion begins as subclinical mineral loss from the enamel and can develop into a visible, early, non-cavitated lesion that may progress to a cavitated caries lesion. Detection of early carious lesions (non-cavitated) and identification of risk factors are crucial for caries prevention and treatment. Untreated dental caries can progress deeper into dentine, eventually reaching the dental pulp and causing pulpal inflammation, which may ultimately lead to pulp necrosis [26,27]. Treatment planning relies on thorough assessment of both the stage of caries lesion development and its activity status. The International Caries Detection and Assessment System (ICDAS) is a standardised clinical scoring system used to detect and classify dental caries [28]. The system categorises lesions into stages of development corresponding to their histological depth within the tooth structure. A numerical scale from 0 to 6 is used, also considering whether the lesions are active or inactive. Code 0 indicates a healthy tooth surface; codes 1 and 2 indicate initial enamel lesions without cavitation; codes 3 to 6 indicate advanced cavitated lesions. Determination of lesion activity is a crucial factor in treatment decisions: active lesions usually require therapeutic intervention (either non-surgical or surgical), while inactive lesions usually require preventive measures such as improved oral hygiene, dietary changes, and fluoride application [28,29].

Comprehensive assessment of the patient’s caries risk profile is essential for establishing a successful treatment protocol. This process presents significant challenges due to the multifactorial aetiology of dental caries, which involves complex interactions between host factors, dietary habits, microbial composition, and salivary parameters that collectively influence the critical balance between demineralisation and remineralisation processes [30]. The Cariogram software (Version 3.0j) serves as an objective tool for caries risk prediction, as it predicts the likelihood of developing new carious lesions based on consumption of sugary foods and beverages, frequency of meals, levels of plaque, *Streptococcus mutans* levels, fluoride use, buffering capacity and salivary flow rate, existing carious lesions and concomitant diseases. It aims to identify patients at high caries risk and facilitate appropriate preventive and therapeutic measures [31].

The literature on dental caries in cGVHD patients is notably scarce [32]. However, it is of great clinical relevance, as studies have shown that these patients have a lower oral health-related quality of life [33,34,35,36,37]. Most studies focus on mucosal changes and alleviation of oral cGVHD symptoms [38,39]. Emerging research explores the role of salivary biomarkers and oral microbial dysbiosis in cGVHD. Proteomic analyses have identified altered expression of immune and tissue maintenance proteins in saliva, reflecting both inflammation and glandular damage [40,41].

This pilot study was prompted by recognition of the important role that cGVHD plays in oral health. Its aim was to assess the impact of cGVHD on salivary caries risk parameters and to investigate the effects of treatment with ECP, cyclosporine, or their combination on these parameters. We hypothesised that cGVHD and its treatment would be associated with altered salivary flow rate, pH of unstimulated and stimulated saliva, buffering capacity of stimulated saliva, and colony density of *Lactobacillus* and *Streptococcus mutans*, potentially contributing to increased caries risk.

## 2. Materials and Methods

This was an observational, single-centre, exploratory cross-sectional pilot study conducted in accordance with the reporting guidelines of the STROBE Statement [42].

Twenty-two adult patients diagnosed with cGVHD as a complication after allo-HSCT were included. The inclusion and exclusion criteria are presented in Table 1 [33].

**Figure 1:**
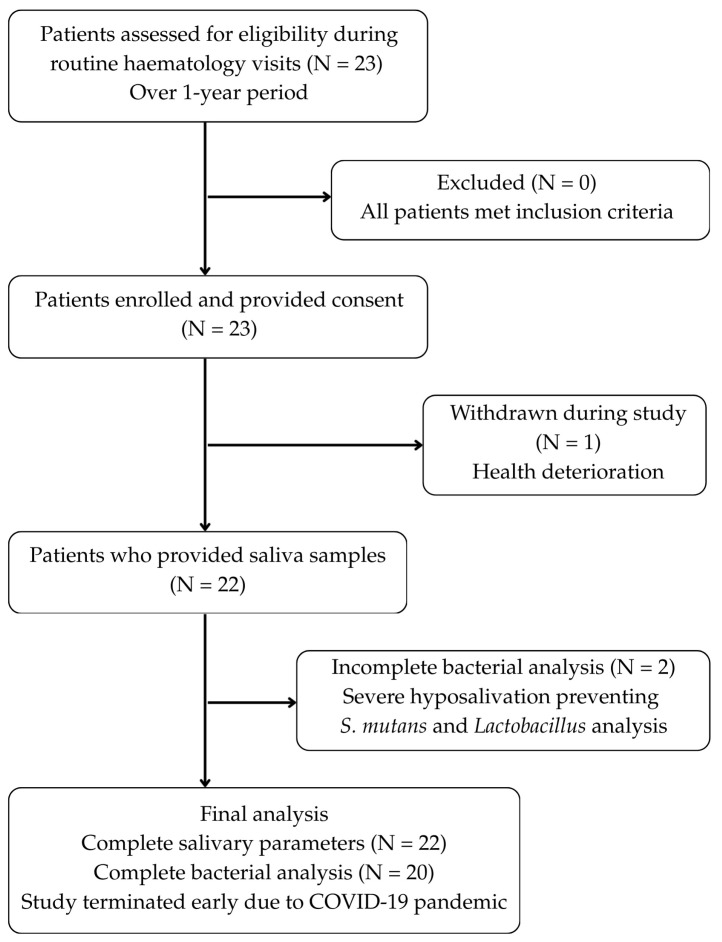
Patient recruitment and selection flowchart.

Figure 1 shows a flowchart of patient recruitment and procedure.

Patients were approached during their scheduled appointments at the Department of Haematology, University Medical Centre Ljubljana, regarding participation in this research. They received comprehensive information about the aims and methodology of the study, including detailed written explanations. Participation was entirely voluntary, and all individuals made an informed decision to participate. Prior to enrolment, each participant formally confirmed their voluntary participation by signing a consent form. The study was approved by the National Medical Ethics Committee of the Republic of Slovenia under number 0120-400/2019/10 on 20 August 2019.

Information from questionnaires on general health, oral health, oral hygiene, and nutrition was collected. The patient’s medication intake was recorded by the haematologist. Salivary samples were collected to assess caries risk factors. Participants were instructed to refrain from eating, drinking, chewing gum, smoking, or performing any oral hygiene procedures for at least one hour before the scheduled clinical examination. If these requirements were not met, saliva collection was postponed. For unstimulated saliva, participants continuously expectorated all saliva produced into a sterile container without swallowing or speaking. To avoid mechanical or sensory stimulation, unstimulated saliva was collected before the intraoral examination. For stimulated saliva, participants chewed a pellet of medical paraffin and expectorated into a labelled container. The collection time was recorded and the salivary flow rate (mL/min) for both unstimulated and stimulated saliva was calculated from the collected volume using a standard medical syringe. This was followed by assessment of dental plaque accumulation on the tooth surfaces. Vario pH device (WTW GmbH, Weilheim, Germany, measuring precision of ±0.01) was used to measure salivary pH. Salivary buffering capacity and bacterial colony densities (*Streptococcus mutans* and *Lactobacillus*) were assessed using semi-quantitative methods (CRT buffer and CRT bacteria tests; Ivoclar Vivadent, Schaan, Liechtenstein). A single dentist, previously trained in ICDAS criteria by senior ORCA (Organisation for Caries Research) experts who are specialists in cariology and restorative dentistry, conducted all clinical assessments [43,44,45]. According to ICDAS criteria, carious lesions were evaluated under standardised lightning, utilising the visual–tactile method with air-drying [28]. To predict the likelihood of new carious lesion formation, the Cariogram software (Version 3.0j) was employed, synthesising data from clinical findings, salivary analysis, and patient questionnaires regarding general health, diet, and oral hygiene [31].

The independent variables were cyclosporine dose and treatment with ECP. The dependent variables assessed were carious teeth, carious tooth surfaces, caries risk, salivary flow rate (unstimulated and stimulated), buffering capacity (stimulated saliva), pH (unstimulated and stimulated saliva), and bacterial colony density (*Streptococcus mutans* and *Lactobacillus*). Data processing and statistical testing were carried out via the SigmaPlot 14.0 package (Systat Software, San Jose, CA, USA). The power analysis determined that a sample size of *n* = 23 was required to detect mean differences in carious teeth and carious tooth surfaces between groups. This calculation was predicated on a statistical power of 0.8, a significance level (α) of 0.05, and a correlation coefficient of 0.5. Results are expressed as means ± standard deviations (SD), with statistical significance defined as *p* < 0.05. To compare differences in the number of carious teeth and carious surfaces, caries risk, pH values, and salivary flow among treatment groups, two-way ANOVA with Bonferroni test was used. To assess differences in bacterial colony densities and buffering capacity between treatment groups ANOVA on ranks was used. To evaluate potential associations between study variables (carious teeth, carious tooth surfaces, salivary flow rates, pH, buffering capacity, bacterial densities, cyclosporine dose, ECP therapy, and caries risk), correlation analysis was performed using Pearson’s coefficient for parametric data and Spearman’s rho for non-parametric data.

## 3. Results

Initially, there were 23 patients, but one participant withdrew due to deteriorating health. Saliva samples were collected from 22 participants. In two of these 22 patients, bacterial colony density of *Streptococcus mutans* and *Lactobacillus* due to severe hyposalivation.

### 3.1. Patient Characteristics and Systemic cGVHD Treatment

The study included 22 patients (10 males, 12 females) with a mean age of 45.05 ± 14.66 years. The mean diet frequency was 5.1 ± 2.0 meals per day, and the mean toothbrushing frequency was 2.4 ± 0.73 times per day.

Primary haematological diagnoses prior to allo-HSCT were acute myeloid leukaemia (*n* = 14), acute lymphoblastic leukaemia (*n* = 6), myelodysplastic syndrome (*n* = 1), and non-Hodgkin lymphoma (*n* = 1). According to the NIH cGVHD Global Rating, disease severity was mild in 15 patients, moderate in 5, and severe in 2.

The results of systemic treatment for cGVHD are presented in Table 2.

### 3.2. Influence of Systemic Therapy on the Measured Parameters

The results concerning the influence of systemic therapy with ECP and/or cyclosporine on the number of carious teeth, carious tooth surfaces, and carious lesions with ICDAS scores 1–2 are presented in Table 3.

There is a statistically significant interaction between ECP and cyclosporine regarding the number of carious teeth (two-way ANOVA, df = 1, F = 12.03, *p* = 0.03). Patients who received both cyclosporine and ECP therapy had fewer carious teeth than those who received only ECP therapy (Bonferroni test, *p* = 0.004). Patients who received only ECP had more carious teeth than those who received neither therapy (Bonferroni test, *p* = 0.008).

There was also a significant interaction between ECP and cyclosporine regarding carious surfaces (two-way ANOVA, df = 1, F = 16.17, *p* < 0.001). Patients who received both cyclosporine and ECP therapy had fewer carious tooth surfaces than those who received only ECP therapy (Bonferroni test, *p* = 0.002). Patients who received only ECP had more carious tooth surfaces than those who received neither therapy (Bonferroni test, *p* = 0.002).

This study showed that systemic therapy with cyclosporine and ECP influenced the number of carious tooth surfaces with non-cavitated lesions (ICDAS 1 and 2) (two-way ANOVA, df = 1, F = 19.99, *p* < 0.001). Patients who received both cyclosporine and ECP therapy had fewer non-cavitated carious tooth surfaces than those treated with ECP alone (Bonferroni test, *p* < 0.001). Patients treated with cyclosporine alone had more non-cavitated carious lesions than those treated with both ECP and cyclosporine (Bonferroni test, *p* < 0.001).

### 3.3. Relationship Between Systemic Factors and Oral Health Parameters

**Figure 2:**
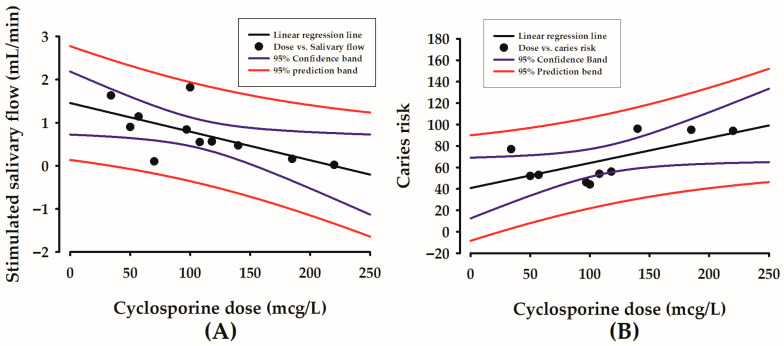
Correlations between cyclosporine dose in mcg/L (X-axis) and stimulated salivary flow in mL/min (Y-axis) (**A**), and between cyclosporine dose in mcg/L (X-axis) and caries risk according to Cariogram in 0–100% (Y-axis) (**B**).

The results of this study showed that a higher cyclosporine dose correlated with lower stimulated salivary flow (R = −0.672, *p* = 0.0486) (Figure 2A), and with higher caries risk according to Cariogram (R = 0.640, *p* = 0.0461) (Figure 2B).

## 4. Discussion

### 4.1. General Findings

This study offers new insights into the complex relationships between cGVHD treatment modalities and oral health parameters, revealing important associations that merit clinical attention and further investigation.

The observed association between higher cyclosporine doses and lower stimulated salivary flow aligns with preclinical evidence indicating that prolonged, high-dose systemic exposure leads to salivary gland toxicity. Previous animal studies have documented histological damage and compositional changes, offering a biological basis for the functional impairment observed in our patients [46,47]. Specific histological findings reported in the literature, such as nuclear irregularities, mitochondrial dysfunction, vacuolisation, and reduced secretory granules, support the hypothesis of a direct cytotoxic mechanism. This structural damage plausibly explains the functional deficit in stimulated salivary flow observed in our cyclosporine-treated cohort [46,47]. Although Spolidorio et al. reported no changes in salivary flow rate in a rat model treated with cyclosporine and tacrolimus, they did observe a significant reduction in total protein content and electrolyte concentrations (Ca^2+^ and Na^+^), accompanied by histopathological alterations [48]. Similarly, Shulman et al. described profound histopathological changes in minor salivary glands, characterised by periductal lymphocytic infiltrate with infiltration and damaged intralobular ducts, fibroplasia in periductal stroma, and mixed lymphocytic and plasmacytic inflammation with destruction of acinar tissue [49]. These findings collectively suggest that cyclosporine compromises salivary gland integrity. Therefore, the positive correlation we observed between cyclosporine dose and caries risk is likely mediated through this drug-induced salivary dysfunction. As salivary flow rate is a critical parameter in caries risk assessment, cyclosporine-associated hyposalivation may represent a key mechanism linking higher drug dosages to increased caries susceptibility [31].

To date, the specific association between cyclosporine dosage and caries development has not been investigated, representing a significant gap in understanding post-transplant oral complications. The development of dental caries as a distinct side effect of allo-HSCT and cGVHD is rarely described in the literature. Where studies exist, they have primarily focused on the negative effects of cGVHD on salivary gland function as the sole potential cause, often overlooking the specific impact of pharmacotherapy [32,50]. For example, Castellarin et al. analysed caries prevalence in a longitudinal cohort and documented a significantly increased incidence of lesions 22 months post-transplantation. Notably, nearly all affected subjects in their study exhibited concurrent salivary dysfunction attributable to cGVHD [32]. Supporting this high risk, Heimdahl et al. reported that 37% of patients developed extensive carious lesions on almost all teeth within one year of transplantation, with a significantly higher incidence in those with a history of acute GVHD [50].

There were no statistically significant differences in the number of carious teeth and tooth surfaces in patients treated with cyclosporine alone. Instead, patients treated with ECP alone had a significantly higher number of carious lesions than those who received both therapies or neither therapy. This pattern suggests that ECP therapy may identify a subgroup of clinically more complex or more severely immunologically affected patients, rather than implying a causal effect of ECP on caries development. Patients undergoing ECP often have more advanced cGVHD or require additional immunomodulation, which may independently predispose them to greater salivary dysfunction or difficulties in maintaining optimal oral hygiene. The finding that ECP-treated patients had a significantly higher number of early non-cavitated lesions supports the idea that ongoing disease activity or treatment burden may contribute to earlier stages of dental demineralisation. Although the results showed differences between therapy groups, we cannot conclude that the type of treatment causes these differences, as no significant associations were found between treatment type and salivary parameters. This discrepancy highlights the likelihood that unmeasured factors, such as baseline caries experience, disease activity, nutritional changes, and treatment-related lifestyle disruptions, may confound the observed associations. The relatively small number of ECP-treated patients further limits definitive interpretation.

Differences in the number of carious lesions may also reflect pre-transplant lesions, the development of cGVHD, and subsequent treatment. Our observation of early, non-cavitated lesions aligns with Castellarin et al., who documented rapid caries onset in previously caries-free patients [32]. The absence of pre-HSCT baseline dental assessments in our study prevented us from distinguishing new from pre-existing disease. Establishing pre-transplant baselines will be crucial in future work to identify true incident lesions and the temporal relationship between immunosuppression, salivary changes, and caries onset. Clinically, these findings emphasise the need for rigorous dental management before and after allo-HSCT. Such vigilant follow-up care allows for early detection and intervention of emerging dental pathologies, including non-cavitated caries lesions, at a stage when the disease process remains reversible. These patients remain vulnerable to caries progression due to immunosuppressive regimens, potential oral cGVHD manifestations, and compromised salivary function.

Furthermore, our literature review highlights the association between cyclosporine and gingival overgrowth, which can physically impede effective oral hygiene [51,52,53,54,55]. This compromised mechanical plaque control, when coupled with the reduced protective capacity of saliva due to hyposalivation, creates a highly cariogenic environment conducive to rapid lesion development. Patients with a more advanced stage of cGVHD may experience more pronounced salivary gland dysfunction, resulting in a significantly reduced salivary flow rate. This may help explain why more carious teeth and tooth surfaces were observed in patients treated with ECP than in those who did not receive ECP therapy. Although we did not find correlations between cGVHD severity and salivary or caries parameters, this may reflect the limited sample size and heterogeneity of disease presentation. It is plausible that micro-level functional salivary impairment precedes clinically measurable changes and would require more sensitive diagnostic techniques for detection.

### 4.2. Strengths and Limitations

The principal strength of this pilot study lies in its clinical significance, as it underscores the importance of dental prevention and treatment for haematological patients undergoing allogeneic haematopoietic stem cell transplantation. It addresses a significant oral health issue, as these patients are greatly affected by the consequences of both the disease and its treatment. The aim of the research was to promote an interdisciplinary approach, involving a haematologist, a dentist, and a patient, to initiate dental planning before transplantation and to continue dental care after transplantation and during immunosuppressive therapy.

This study has several limitations that should be acknowledged. The cross-sectional design precludes the establishment of causal relationships between treatment modalities and salivary parameters, allowing only the assessment of associations at a single time point. Selection bias may have occurred, as treatment assignment was not randomised but based on clinical indications, potentially confounding the relationship between treatment modality and salivary parameters.

A major challenge in this research was the timing of the COVID-19 pandemic. Restrictions in the healthcare system led to fewer transplantations and reduced participant adherence to therapy. Although the planned sample was initially recruited, the study was terminated early due to the pandemic, resulting in participant dropout and incomplete data collection. Consequently, the final sample size was limited, which may have reduced the statistical power of the analyses and the generalisability of the findings. However, sample size limitations are common in cGVHD research due to the relatively rare nature of the condition, with several published studies reporting even smaller cohorts, some including fewer than 10 patients.

A significant limitation is the absence of pre-treatment baseline measurements of salivary parameters and caries risk factors. Without knowledge of patients’ oral health status prior to cGVHD development and treatment initiation, it is not possible to determine whether the observed alterations in salivary function and caries risk are attributable to cGVHD and its treatment or reflect pre-existing conditions. Additionally, reliance on self-reported questionnaire data for general health, oral health, oral hygiene, and nutritional information may introduce reporting bias, as patients may provide socially desirable responses or have varying perceptions of symptom severity.

Furthermore, potential confounding factors such as cGVHD severity, time since transplantation, concomitant medications, and individual patient characteristics were not fully controlled in the analysis. The varying duration of treatment and time since transplantation among participants may have influenced salivary parameters, but these temporal factors were not systematically evaluated. Salivary measurements represent single time-point assessments and may not reflect the dynamic nature of salivary function throughout the day or disease progression. In addition, the use of semi-quantitative microbiological methods may limit analytical precision compared with more advanced quantitative techniques. Finally, as a single-centre study, the generalisability of these findings to other populations or treatment protocols may be limited.

To address these methodological limitations, we have designed a larger prospective study that will follow patients from their pre-transplantation baseline through their post-transplantation journey. This study will systematically document caries risk factors and comprehensively assess variations in oral health parameters during the transplantation process. With the collaboration of haematologists and our methodological approach, we aim to improve understanding of caries progression patterns while facilitating the development of evidence-based preventive measures. The identified risk factors will form the basis for establishing treatment protocols aimed at minimising the incidence of caries and maintaining oral health integrity in this medically compromised population.

## 5. Conclusions

This exploratory cross-sectional pilot study identified significant associations between cGVHD treatment modalities and salivary caries risk factors. Higher doses of cyclosporine were associated with reduced salivary flow, while ECP therapy was linked to a greater number of carious lesions. Although the small sample size limits statistical power and these associations require confirmation in larger longitudinal cohorts, these findings underscore the need for an interdisciplinary approach and systematic monitoring to ensure effective caries prevention in patients with cGVHD. Emphasis should be placed on the early detection of non-cavitated, reversible caries lesions, where timely preventive management can successfully halt disease progression.

## Figures and Tables

**Table 1:** Inclusion and exclusion criteria for the study.

Inclusion Criteria	Exclusion Criteria
The diagnosis of cGVHD	Presence of less than 3 teeth per quadrant
Presence of at least 3 teeth per quadrant	The use of removable denture

**Table 2:** Systemic treatment of cGVHD (*n* = 22).

Therapy	Cyclosporine Treatment Period	Cyclosporine Dose	ECP Treatment Period	ECP Weekly Frequency
	8.17 ± 8.6 months	115.92 ± 62.3 mcg/L	16.71 ± 16.6 months	1.2 ± 0.8 times per week

**Table 3:** Means and standard deviations of carious teeth, carious tooth surfaces, and carious lesions with ICDAS scores of 1–2 in patients with different therapy regimens (*n* = 22).

	Cyclosporine (*n* = 8)	ECP (*n* = 3)	Cyclosporine + ECP (*n* = 4)	Neither Cyclosporine Nor ECP (*n* = 7)
Carious teeth	9.1 ± 1.8	16.0 ± 3.0	3.2 ± 2.6	5.4 ± 1.9
Carious surfaces	14.9 ± 3.1	28.7 ± 5.0	3.7 ± 4.4	7.3 ± 3.3
ICDAS 1–2	6.1 ± 1.9	19.3 ± 3.2	0.7 ± 2.8	1.1 ± 2.1

## Data Availability

The raw data supporting the conclusions of this article will be made available by the authors on request.
